# Accidental Use of Milk With an Increased Concentration of Aflatoxins Causes Significant DNA Damage in Hospital Workers Exposed to Ionizing Radiation

**DOI:** 10.3389/fpubh.2020.00323

**Published:** 2020-07-24

**Authors:** Jasminka Mrdjanovic, Jan Sudji, Branislava Srdjenovic, Sanja Dojcinovic, Visnja Bogdanovic, Dea Karaba Jakovljevic, Vladimir Jurisic

**Affiliations:** ^1^Faculty of Medicine, Oncology Institute of Vojvodina, University of Novi Sad, Sremska Kamenica, Serbia; ^2^Institute of Occupational Health, Novi Sad, Serbia; ^3^Department of Pharmacy, Faculty of Medicine, University of Novi Sad, Novi Sad, Serbia; ^4^Department of Physiology, Faculty of Medicine, University of Novi Sad, Novi Sad, Serbia; ^5^Faculty of Medical Sciences, University of Kragujevac, Kragujevac, Serbia

**Keywords:** aflatoxin, genotoxicology, ionizing radiation, occupational exposure, 8-OHdG, hospital workers, ELISA, micronuclei

## Abstract

The occupational exposure to ionizing radiation (Irad) or associated with mycotoxin-contaminated food may lead to genome damage and contribute to health risk. DNA damage in 80 blood samples of hospital workers occupationally exposed to low—doses of Irad was compared with 80 healthy controls. Among them, 40 participants accidentally consumed milk with increased concentration of Aflatoxin. All participants underwent the testing for micronuclei from blood, and 40 of them 8-OHdG from urine. The frequency of micronuclei (MN) was analyzed by cytokinesis-block peripheral blood lymphocytes and the level of urinary 8-hydroxy-2′-deoxyguanosine (8-OHdG) by ELISA. The Irad led to increased frequency of MN (*p* < 0.05) and 8-OHdG level at exposed hospital workers. The consumption of milk with increased concentration of aflatoxin probably raised MN frequency and 8-OHdG value. Higher consumption of aflatoxin-contaminated milk (≥2 L/monthly) caused significantly increased MN frequency and 8-OHdG value in comparison to lower milk intake (≤0.5 L/monthly). Also, confounding factors, such as age, gender, and smoking status of all participants were included in the study. The obtained results revealed an increased incidence of MN and 8-OHdG level among hospital workers exposed to low-doses of IRad and milk with increased aflatoxin concentration.

## Highlights

- Determination of DNA damage among hospital personnel after accidental consumption of milk in the period characterized by increased concentration of aflatoxin.
- Increased frequency of micronuclei and the level of 8-OHdG were noticed in participants exposed to Irad and aflatoxin-contaminated milk.
- Consumption of milk with increased aflatoxin content contributes to DNA damage.

## Introduction

Ionizing radiation (IRad) is a major stress factor that may induce cell damage and, consequently, carcinogenesis (1). IRad acts directly or indirectly via radiolysis of water, thereby creating a reactive oxidative species (ROS). ROS can attack nucleic acids, which is followed by many different types of DNA damages. DNA damage may occur as a result of the impact of IRad oxygen radicals generated during the endogenous process, as well as those from working and living environment. Therefore, it is very important to assess the absorbed dose of IRad in persons who are occupationally exposed to IRad in compliance with relevant legislation.

In combination with the occupational exposure to IRad, eating habits during particular period of life may also contribute to additional genome damage. Consuming food containing high levels of mycotoxins, which are associated with certain disorders in both humans and animals, causes significant health risk.

Aflatoxins (AFT), well-known mycotoxins and highly toxic metabolites are produced by *Aspergillus* species, mainly *A. flavus* and *A. parasiticus* (2). According to the International Association for Research on Cancer (IARC) classification, AFB_1_, AFB_2_, AFG_1_, and AFG_2_ are group 1 carcinogens, whereas AFM_1_ is a group 2B carcinogen (3).

The AFB1, known DNA-damage agent, bonds covalently its metabolites (AFB1-−8,9-epoxide) to DNA in target cells, which results in AFB1-N7-guanine adducts, consequently leading to mutations and tumorous changes at different organs (4).

In terms of cytotoxic effect, AFB1 leads to lipid peroxidation and oxidative stress in hepatocytes and inhibits nucleotide phosphodiesterase cyclical activity in tissues (5). Aflatoxin-B1 and -M1, its main monohydroxilated metabolite, enter into the body of domestic animals through contaminated plant food and milk products consequently become an indirect source of AFTs.

The majority of developed countries established maximum legally permitted residue levels (MRL) for AF M1 in milk. In the European Union, MRL for AF M1 is 0.05 μg/kg milk or milk-based product (6). In Serbia, MRL for AFM1 (7) is harmonized with the values set out by European Union (EU) Regulation.

The published studies from Mediterranean and Middle East countries indicate that environmental conditions, especially warm and dry weather, may favor the occurrence of AFTs in agricultural products and therefore AF M1 in milk (8). Low contamination frequency of AF M1 in the reports from Serbia for the period before AFT contamination can be explained by the lower rate of AFTs in maize and other feed material in Serbia due to optimal weather conditions in these years (8).

The extreme warm and dry weather conditions in 2012 led to contamination of agricultural crops and elevation of AFT B1 in animal feed used for feeding lactating animals in Serbia and the region- almost 80% of the total production of maize in 2012 was estimated to be affected by aflatoxins, which caused large-scale crisis in February 2013, when AFM1 was detected in milk produced by Serbian dairy companies (9, 10).

Two additional studies indicated serious risk to consumers since AF M1 levels exceeding EU MRL (at concentration range from 0.05 to 0.98% μg/kg) were detected in 80–100% of different milk samples collected between February and June 2013 on the Serbian market (11, 12).

Therefore, taking into account geno- and cytotoxic properties of AF M1, milk consumption as a monitored parameter was also included in our investigation.

During the last decade, the Cytokinesis-Blocked Micronucleus Assay (CBMN) has become a thoroughly validated and standardized technique for the evaluation of DNA damage at individuals occupational, medical and accidentally exposed to radiation. It is known that increased MN frequency in peripheral blood lymphocytes represents a predictive biomarker of cancer risk (13). As a part of nutrigenetics, CBMN assay is used to determine the influence of dietary habits on the changes in the human genome (14).

Also, 8-OHdG, the repair product of excision enzymes which is excreted through urine is used as a biomarker to assess the extent of oxidative DNA damage and repair in the occupational setting (15).

To date, there is no information on conducting risk assessment for two agents with different mechanisms of action (i.e., energy deposition from ionizing radiation vs. DNA interactions with chemicals). In our investigation, we examined the influence of low-dose IRad and consumption of milk in the period when over 80% samples from Serbian market had aflatoxin concentrations >0.05 μg kg^−1^ on the aforementioned biomarkers of DNA damage. Our study was conceived to monitoring similar biological endpoints for determining genetic hazard, micronuclei in peripheral blood and 8-OHdG in urine. The analysis was conducted on medical workers chronically exposed to IRad and the results were compared with the unexposed control groups. The subjects were also divided into the sub-groups according to age, gender and smoking status. The combination of monitored biomarkers could give more complete view of the influence of both IRad and consumption of AF M1-contaminated milk on occupationally exposed persons and thus provide information about their cumulative health risk associated with carcinogenesis.

## Experimental Procedures

### Group Description

This retrospective study included 160 participants-−80 healthy volunteers and 80 hospital workers chronically exposed to low doses of ionizing radiation, employed at the Oncology Institute of Vojvodina and the Institute for Lung Diseases of Vojvodina, Republic of Serbia. Various equipment was the source of occupational radiation exposure among bronchoscopy and radiotherapy medical personnel. The workers in bronchoscopy unit performed, wearing lead aprons, up to 10 interventions per day, and X-ray source was active to 2 min during bronchoscopy procedure, and the ones from radiotherapy unit were in a control room, protected from direct source of ionizing radiation. Persons who had medical treatment, radiography, or vaccination within the previous 9 months were not included in the study. The questionnaire filled by each participant included general information about professional exposure to Irad and about life habits like smoking, alcohol consumption, medical history, drug intake and diagnostic medical irradiation. Blood samples from all participants were collected during 2012, until June 2013. In addition to blood samples for micronuclei test during the first half of 2013, urine samples were collected for 8-OHdG, and participants filled in the questionnaire on habits regarding milk consumption. The study was approved by the Ethics Committee of the Institute for Lung Diseases of Vojvodina and informed consent was obtained from participants.

### Cytokinesis Block Micronucleus Test (CBMN)

Heparinized whole blood was collected by venous puncture from participants and used for the peripheral blood lymphocyte cultures in CBMN test. Briefly, 0.5 ml of the whole blood was added to 5 ml of RPMI 1640 cell culture medium (Sigma, USA) supplemented with 2 mM glutamine, 20% of heat-inactivated fetal calf serum (FCS, NIVNS, Serbia) and antibiotics: 100 IU/ml penicillin and 100 μg/ml streptomycin (ICN, Serbia). Cell cultures were stimulated for division with phytohemagglutinin (PHA-M, Sigma, USA) at a final concentration of 20 μg/mL and incubated at 37°C for 72 h in 5% CO_2_ atmosphere with 95% humidity.

CBMN was performed applying standard cytogenetic procedure with minor modifications regarding staining (1). Forty-four hours after stimulation of the lymphocyte culture with PHA, cytochalasin-B (Sigma, USA) was added at final concentration of 6 μg/mL. After 72 h, the cells were collected by centrifugation, exposed to a cold 0.075 M KCl hypotonic solution and fixed three times. The first fixation was in methanol-acetic acid (3:1) with 1% formaldehyde, where the two following fixations were in methanol—acetic acid (3:1) alone. Drops of a concentrated cells suspension were placed on dried slides. Cells were stained with Giemsa (2%) in distilled water with three drops of NH_4_OH for 9 min.

At least 1,000 cells per each sample were analyzed. Monitored values included: frequency of micronuclei, micronucleus distribution and proliferation index. MN frequency was presented as a number of micronuclei per 1,000 examined binuclear cells. Micronucleus distribution was acquired by scoring the binuclear cells containing one or more micronuclei. The proliferation index (PI), which represents a measure of the number of cell cycles that a cell population passes through, was calculated according to the formula:

$$\begin{array}{l} \text{NDI}=\text{M}1+2\text{M}2+3\left(\text{M}3+\text{M}4\right)/\text{N} \end{array}$$

where M1–M4 represents the numbers of cells with 1–4 nuclei, respectively, and *N* is the total number of scored cells (1). The prepared material was observed and analyzed by light microscopy (Olympus BX51, Germany).

### 8-Hydroxy-2′-Deoxyguanosine (8-OHdG)

Determination of 8-OHdG level was conducted according to the commercial enzyme-linked immunosorbent assay kit (highly—sensitive 8-OHdG check; Japan Institute for the Control of Aging, Nikken SEIL Co., Ltd., Shizuoka, Japan). The urinary concentration of 8-OHdG was expressed by creatinine to avoid the effect of urine volume fluctuation [8-OHdG (μg/ml): creatinine (g/ml)] = 8-OHdG (μg/g creatinine). For the determination of urinary creatinine concentrations a modified Jaffe's method was used (16).

### Statistics

To obtain the differences between the observed groups relative to micronuclei frequency and 8-OHdG values, the results were analyzed by Wilcoxon Matched Pairs Test, Mann Whitney U test and ANOVA, using STATISTICA Release 12. After adjusting for potential confounding factors (age, gender, and smoking status), multivariate analysis was performed by ANCOVA to assess the differences in micronuclei and 8-OHdG among the study groups. Adjustment for multiple testing was carried out by a *post-hoc* LSD test. The statistical significance for all tests was set at *p* < 0.05.

## Results

### The Characteristics of the Subjects Included in the Study

The characteristics of both control and the group exposed to Irad are shown in Table 1. Regarding to the period of sample collection and milk consumption habits, both groups are divided to sub-groups: “with AFT”-participants who consumed the milk with increased concentration of AFT and “without AFT”-participants who didn't consume the milk with increased concentration of AFT.

**Table 1:** Characteristics of subjects included in the study.

**Variables**		**Control (*****n*** **=** **80)**		**Irad (*****n*** **=** **80)**	
		**Without AFT**	**With AFT**	**Without AFT**	**With AFT**
		**(*n* = 70)**	**(*n* = 10)**	**(*n* = 50)**	**(*n* = 30)**
Age (years)	Median	36	44	42	46.5
	Range	22–68	26–65	24–61	28–62
Young	22–45 years	47	5	30	14
Old	46–62 years	23	5	21	16
Gender	Male	27	2	21	4
	Female	43	8	29	26
Exposure time to ionizing radiation	Median	nn	nn	na	16.50
(years)	Range	nn	nn		2–36
Smoking status (%)	Non-smokers	52.85	80	21	63
	Smokers	47.14	20	29	37
Duration of smoking (years)	Median	na	16	na	26
	Range		7–25		10–39
Nutrition (%)	Vegetarian	0	0	0	0
	Non-vegetarian	100	100	100	100
Milk consumption	≥2 L/month	na	6	nn	19
(Number of individuals)	≤ 0.5 L/month		4		11
Irradiation during last month (mSv)	Median	nn	nn	na	0.21
	Range	nn	nn		0.16–2.05

*Na, not available; nn, not necessary*.

### Effect of Ionizing Irradiation on Micronuclei and 8-OHdG in Hospital Workers

**Figure 1:**
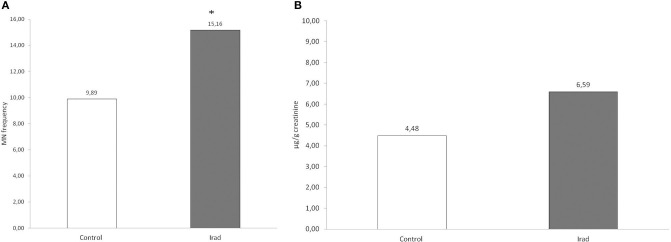
Micronucleus frequency **(A)** and 8-OHdG values **(B)** in hospital workers exposed to ionizing irradiation. 8-OHdG, 8-hydroxy-2′-deoxyguanosine value; Control, unexposed healthy voluntairs; Irad, hospital workers exposed to Irad.

The analysis of micronuclei (Figure 1A) and 8-OHdG (Figure 1B) revealed that hospital workers exposed to ionizing irradiation had higher values than unexposed healthy voluntairs from the control group. The difference between MN frequency in a group exposed to Irad and control group were significant (MN: *p* < 0.05; Wilcoxon test) (Figure 1A).

### The Effects of Milk Consumption on Micronuclei and 8-OHdG

In both control group and the group exposed to Irad, participants who consumed a milk with AFT had higher frequency of micronuclei than those who didn't use contaminated milk.

**Figure 2:**
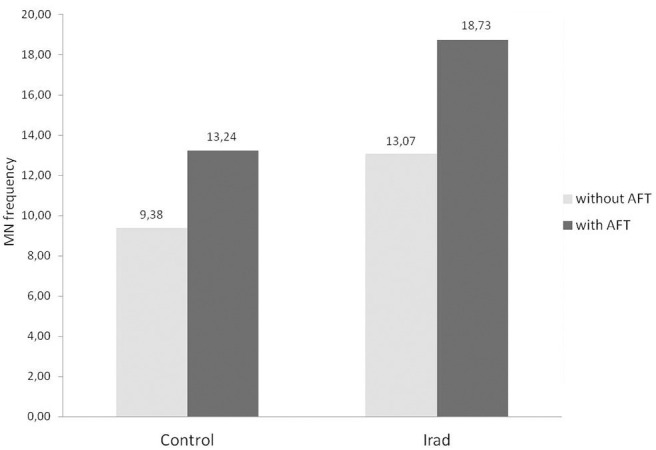
Micronucleus frequency in relation to milk consumption in hospital workers exposed to ionizing irradiation. Control, unexposed healthy voluntairs; Irad, hospital workers exposed to Irad; ^*^, statistically significant difference in comparison to Irad without AFT; †, statistically significant difference in comparison to control without AFT.

Statistical analysis revealed that MN frequency in workers exposed to Irad who consumed milk with AFT was significantly higher in comparison to workers who didn't (*p* = 0.015; *post-hoc* LSD test), as well as in comparison to unexposed volunteers who also didn't use contaminated milk (*p* = 0.006; *post-hoc* LSD test) (Figure 2).

The groups “with AFT” were divided into two sub-groups according to the amount of consumed milk during the previous 3–6 months—a group that consumed <0.5 L milk/month and a group that consumed more than 2 L milk/month. The number of subjects in each sub-group is shown in Table 1.

**Figure 3:**
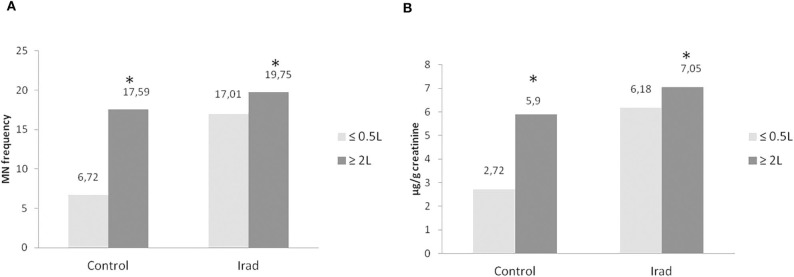
The micronucleus frequency **(A)** and 8-OHdG values **(B)** in relation to different amounts of consumed milk with AFT in hospital workers exposed to ionizing irradiation. MN, micronucleus frequency; 8-OHdG, 8-hydroxy-2′-deoxyguanosine value; ^*^, statistically significant difference in comparison to the control group, who consumed ≤0.5 L/month of milk.

The MN frequency (Figure 3A) and 8-OHdG (Figure 3B) values in subjects from control groups who consumed more than 2 L milk/month were significantly higher (*p* < 0.05; Mann Whitney U test) in comparison to controls who consumed ≤0.5 L/month of milk. Hospital workers, occupationally exposed to ionizing radiation, who consumed more than 2 L milk/month, had also significantly higher MN frequency and 8-OHdG values (*p* < 0.05; Mann Whitney *U* test) as compared with subjects from control group.

### Effect of Age, Gender, and Smoking Status on Micronuclei and 8-OHdG

The participants were divided into two sub-groups according to the age: younger (<45 years) and older (>45 years). The number of younger and older subjects in the control and exposed groups is shown in Table 1.

**Figure 4:**
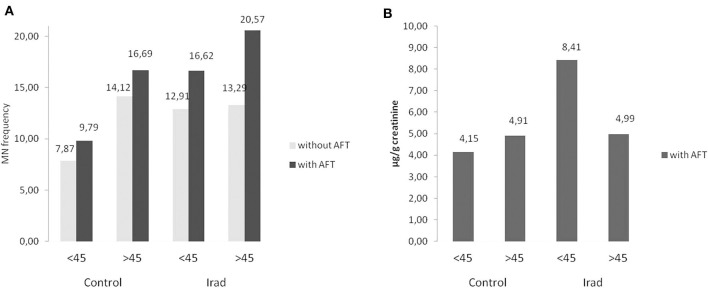
Influence of participants' age on micronucleus frequency **(A)** and 8-OHdG values **(B)** in hospital workers exposed to ionizing irradiation. MN, micronucleus frequency; 8-OHdG, 8-hydroxy-2′-deoxyguanosine value.

In all groups, older subjects had higher MN frequency compared to younger (Figure 4A) as well as in control group regarding to 8-OHdG values (Figure 4B).

**Figure 5:**
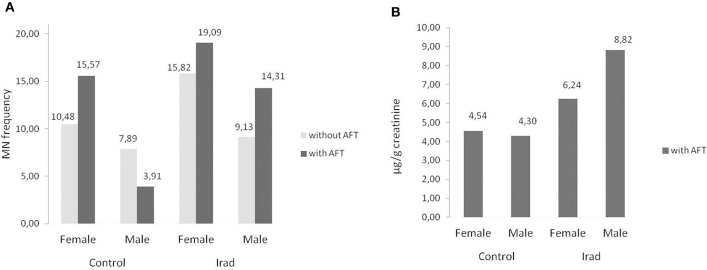
Influence of participants' gender on micronucleus frequency. Effects on micronuclei frequencies **(A)** and on 8-OH values **(B)** in hospital workers exposed to ionizing irradiation. MN, micronucleus frequency; 8-OHdG, 8-hydroxy-2′-deoxyguanosine value.

As related to the gender, females had higher MN frequency than males in both control group and group exposed to Irad (Figure 5A) but this is not case for 8-OHdG values (Figure 5B).

**Figure 6:**
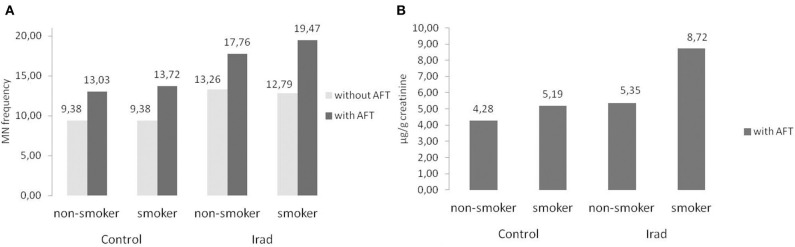
Influence of participants' smoking status on micronucleus frequency **(A)** and 8-OHdG values **(B)** in hospital workers exposed to ionizing irradiation. MN, micronucleus frequency; 8-OHdG, 8-hydroxy-2′-deoxyguanosine value.

Smokers from both groups had higher MN frequency (Figure 6A) and 8-OHdG values (Figure 6B) in comparison to non-smokers (Table 2).

**Table 2:** Multivariate analysis of the influence of age, gender, and smoking status on micronuclei and 8-OHdG.

**Variables**	**ANCOVA (*****p*****-values)**	
	**MN frequency**	**8-OHdG**
Irad with AFT/control without AFT	0.006^*^	–
Irad with AFT/Irad without AFT	0.015^*^	–
Control with AFT/control without AFT	0.543	–
Irad with AFT/control with AFT	0.246	0.535
Age	0.520	0.77
Gender	0.084	0.572
Smoking status	0.138	0.214

* *p < 0.05 was considered as statistically significant*.

ANCOVA showed significant differences in MN frequency (*F* = 2.786, *p* = 0.043) among the groups. The confounding factors- age (*F* = 0.416, *p* = 0.520), gender (*F* = 3.021, *p* = 0.084), and smoking status (*F* = 2.222, *p* = 0.138), were not related to the differences in MN frequency among the study groups. However, regarding to 8-OHdG values, ANCOVA showed no differences (*F* = 0.393, *p* = 0.535) among the groups. Also, the age (*F* = 0.518, *p* = 0.477), gender (*F* = 0.326, *p* = 0.572), and smoking status (*F* = 0.470, *p* = 1.607, η = 0.214), were not related to 8-OHdG levels among the groups.

## Discussion

This study showed that hospital workers occupationally exposed to IRad have increased values of micronuclei and 8-OHdG, which indicates DNA damage (Figure 1). Moreover, it can be assumed that the accidental consumption of milk in a period when AF M1 level was elevated in the majority of samples available on the market (over 86% of samples exceeded the maximum level of 0.05 mg/kg set by EU (9) contributes to an increase in values of the examined biomarkers in both control and group of subjects exposed to IRad.

It is well-known that the biological effect of the radiation is manifesting through direct and indirect DNA damage. Thereby, oxidative DNA damage by ROS exceeds the direct effect of the IRad (17). As a result of elevated ROS, transcription factors and their corresponding genes are permanently activated, which, coupled with increased DNA damage, creates the environment for the occurrence of malignant phenotype (18).

In recent years, evaluation of micronuclei became a powerful, well-accepted method in radiation biodosimetry especially for determining level of DNA damage in subjects for whom a pre-exposure analysis is not available (19) as in the case of workers exposed to IRad in our study.

The group of workers in bronchoscopy and radiotherapy units in our study had significantly higher micronucleus frequency indicating elevated genome damage due to long exposure to low doses of IRad. Also, none of the workers were accidentally irradiated, so only the cumulative effect of IRad was monitored. Results obtained in this study are in agreement with our previous study about occupational exposure to IRad (20). According to Fenech et al., increased frequency of MN is manifested by the appearance of acentric fragments and dicentric chromosomes characteristic for IRad influence, but it also provides an insight into miss-repaired DNA breaks reduced DNA reparation in our group of IRad exposed subjects (21). Radiation-induced chromosome aberrations, such as MN are the result of non-homologous end joining repair pathway, responsible for unrepaired or miss-repaired double strand breaks of DNA (22). Taking this into account, the increased MN values obtained in this study could also be the result of less efficient DNA reparation in IRad exposed group.

It is known that early biochemical changes occur immediately upon the exposure of cells to IRad. Since reactive oxygen (ROS) and nitrogen species (RNS) are continually being generated, oxidative changes happen to continue even months after the initial exposure (23). ROS and RNS, whose main generators are radiolysis of water and early activation of nitric oxide synthases under ambient oxygen can attack DNA resulting in several alterations, including DNA breaks, base damage, destruction of sugars, cross-links, and telomere dysfunction (24). The oxidized nucleosides and bases are generally excreted into urine and the base-excision repair pathway takes part in their genesis. So, based on these considerations, 8-OHdG detected in urine has been described as a sensitive marker to evaluate oxidative DNA modification (25). From a methodological standpoint, a combination of the MN with other tests, such as 8-OHdG, which measure oxidative DNA damage, can provide additional useful information.

In spite of the limitations of our study (we had restricted number of participants who consumed AFT-contaminated milk) we consider that combining indicators of DNA damage within genotoxic monitoring could give us impactful assessment of potential cumulative effect both AFT and Irad.

The group exposed to Irad had higher values of 8-OHdG in comparison to the control group, but without statistical significance. This result corresponds with MN frequency and additionally confirms that occupational IRad exposure of professional and medical staff contributes to higher DNA damage.

Also, we presume that the absence of statistical significance for 8-OHdG values between IRad exposed and control group in our study, contrary to the significant difference obtained by MN assay, could be explained by the small number of subjects in the experimental group.

In addition, the fact that urinary 8-OHdG levels may be influenced by many factors, among which is polymorphic *hOGG1* genotype that causes inter-individual variability in 8-OH-Gua repair and has a major role in the prevention of ROS-induced carcinogenesis, should not be overlooked (15). Clearly understanding of DNA repair participant phenotypes, which exceed the purpose of this study, could give more precise answers on these questions.

During 2013, throughout collection and investigation of blood and urine samples for MN and 8-OHdG in this investigation, original literature data reported that 86% cow milk samples from Serbian market contained AF M1 at concentration higher than the approved maximum residue levels (MRL) (9). According to Jajić et al., in all 4 groups of samples from 2013 [pasteurized, raw, ultra-high temperature treated (UHT), and organic milk], very high levels of AF M1 contamination were established ranging from 80 to 100% (11).

The results of this study revealed that subjects who accidentally consumed AFT-contaminated milk had higher MN frequencies as compared to the controls (Figure 2). In the group of IRad exposed subjects who consumed AFT-contaminated milk, the MN frequency was significantly higher in comparison to IRad exposed ones without AFT-contaminated milk consumption. These results speak in favor that DNA damage effect is might be due to AF M1 influence.

Both of used tests confirmed that participants who consumed more AF M1 milk had more pronounced DNA damage than the ones who consumed less milk (Figure 3). The results of this study showed that consumption of higher amounts of milk in the period of AF M1 contamination corresponds with increased values of examined DNA damage biomarkers, which is in accordance with published results (5). Also previous study from 2005 which included participants who didn't consumed AFT-contaminated milk revealed lower frequency of MN in both control group and group exposed to IRad as compared to actual results (20). This difference in the degree of micronuclei frequency supports the notion about aflatoxin co-influence on DNA damage presented in this study. The cumulative effect of both AFT in milk and IRad damaging effect could be the cause of significantly increased values of MN frequency.

Concerning to confounding factors, our study showed that the effect of age is reflected in elevated MN frequency in older subjects in both control and exposed group (Figure 4A), which is in agreement with our previous studies (26, 27). The explanation of this phenomenon, as seen in other studies, probably lies in a combination of factors, such as cumulative effect of acquired mutations in genes involved in DNA repair chromosome segregation and cell cycle checkpoint, as well as aberrations in chromosomes caused by exposure to endogenous genotoxins and exposure to environmental or occupational genotoxins (28).

In regard to gender, the results of this study confirmed that females had higher MN frequency than males in both control group and the group exposed to Irad (Figure 5A). This phenomenon can be explained by a random loss of an X chromosome, which is eliminated from the nucleus to form a micronucleus (29). It reflects the importance of the gender as a variable in studies utilizing the cytokinesis-block micronucleus assay as a biomarker of chromosome damage. Similare to this results, previous studies have shown also that gender and age are not associated with changes in 8-OHdG level (30). The evaluation of influence of smoking as a confounding factor revealed that smokers in both groups had higher MN frequency and 8-OHdG values in comparison to non-smokers (Figure 6). Similarly, several studies showed somewhat higher 8-OHdG levels among smokers (31, 32). Since smoking causes DNA damage it has to be taken into account as a cofactor when assessing the risks of combined exposures to Irad and AF M1.

The analysis of individual confounding factors showed their slight influence on the frequency of micronuclei. However, multifactorial analysis showed that these factors were not related to DNA damage measured by 8-OHdG and micronuclei tests and also pointed out DNA damage induced by the cumulative effect of Irad and consuming AFT-milk.

## Conclusion

This study shows increased genome damage in hospital workers occupationally exposed to low-dose ionizing radiation detected using MN and 8-OHdG assay.

Additionally, this study indicates that accidental consumption of milk with elevated aflatoxin concentrations might contribute to increased values of both investigated DNA damage biomarkers. Regardless of the limitations of our study we consider that combining indicators of DNA damage could give us impactful assessment of potential cumulative effect both AFT and Irad. It is justified to assume that co-exposure to Irad and AFT could increase health risk in occupationally exposed personnel, which point out the necessity of health risk assessment. Further investigations are required in order to mor5e closely reveal the cumulative effect of exposure to mixed radiation/chemical agents with different action mechanisms, i.e., to provide additional information about health risk of carcinogenesis in relation to eating habits.

## Data Availability Statement

The datasets presented in this article are not readily available to protect participant identity. Requests to access the datasets should be directed to the corresponding author.

## Ethics Statement

The studies involving human participants were reviewed and approved by Institute of Oncology Vojvodina, Novi Sad, Serbia. The patients/participants provided their written informed consent to participate in this study.

## Author Contributions

JM did the analysis and wrote the concept paper. JS approved the patients from the clinic. BS and SD did the part of the analysis. DJ helped with the analysis. VJ formulated the concept and corrected and approved the final version of the paper, as project chief. All authors contributed to the article and approved the submitted version.

## Conflict of Interest

The authors declare that the research was conducted in the absence of any commercial or financial relationships that could be construed as a potential conflict of interest.

## Abbreviations

OHdG: 8-hydroxy-2′-deoxyguanosine
AFT: aflatoxin
CBMN: Cytokinesis-Blocked Micronucleus Assay
DNA: Deoxyribonucleic acid
ELISA: The enzyme-linked immunosorbent assay
FCS: fetal calf serum
IARC: International agency for research on cancer
IRad: ionizing radiation
MN: micronuclei
MRL: maximum residue level
RNS: Reactive nitrogen species
ROS: reactive oxygen species.

## References

[1] International Atomic Energy Agency Cytogenetic Analysis for Radiation Dose Assessment: A Manual. Technical report series no. 405. Vienna: IAEA (2001).

[2] O'Riordan MJ Wilkinson MG A survey of the incidence and level of aflatoxin determination in a range of imported spices preparations on the Irish retail market. Food Chem. (2008) 107:1429–35. 10.1016/j.foodchem.2007.09.073

[3] IARC Monographs on the Evaluation of Carcinogenic Risks to Humans: Chemical Agents and Related Occupations. A Review of Human Carcinogens. Lyon: IARC (2012). PMC4781612 23189753

[4] Bailey GS Role of aflatoxin-DNA adducts in the cancer process. In: Eaton DL Groopman JD, editors. The Toxicology of Aflatoxins: Human Health, Veterinary, and Agricultural Significance. San Diego, CA: Academic Press (1994). p. 137–48. 10.1016/B978-0-12-228255-3.50012-X

[5] Bonsi P Agusti-Tocco G Palmery M Giorgi M. Aflatoxin B1 is an inhibitor of cyclic nucleotide phosphodiesterase activity. Gen Pharmacol. (1999) 32:615–9. 10.1016/S0306-3623(98)00282-1 10382866

[6] European Commission Commission Regulation No. 1881/2006 of December 19th setting maximum levels of certain contaminants in foodstuffs. Off J Eur Union. (2006) L364:5–24.

[7] Serbian Regulation Maximum allowed contents of contaminants in food and feed. Off Bull Republ Serbia. (2015) 84:1.

[8] EFSA Report for 2010 on the results from the monitoring of veterinary medicinal product residues and other substances in live animals and animal products. EFSA J. (2012) 212:1–64. 10.2903/sp.efsa.2012.EN-212

[9] Kos J Mastilović JE Hajnal B Natural occurrence of aflatoxins in maize harvested in Serbia during 2009–2012. Food Control. (2013) 34:31–4. 10.1016/j.foodcont.2013.04.004

[10] Škrbić B Živančev J Antić I Godula M Levels of aflatoxin M1 in different types of milk collected in Serbia: assessment of human and animal exposure. Food Control. (2014) 40:113–9. 10.1016/j.foodcont.2013.11.039

[11] Jajić I Glamočić D Krstović S Polovinski Horvatović M Aflatoxin M1 occurrence in Serbian milk and its impact on legislative. J Hell Vet Med Soc. (2018) 69:1283–90. 10.12681/jhvms.19618

[12] Tomašević I Petrović J Jovetić M Raičević S Milojević M Miočinović J Two year survey on the occurrence and seasonal variation of aflatoxin M1 in milk and milk products in Serbia. Food Control. (2015) 56:64–70. 10.1016/j.foodcont.2015.03.017

[13] Bonassi S Neri M Lando C Ceppi M Lin YP Chang WP. The HUMN collaborative group, effect of smoking habit on the frequency of micronuclei in human lymphocytes: results from the Human MicroNucleus Project. Mutat Res. (2003) 543:155–66. 10.1016/S1383-5742(03)00013-9 12644185

[14] Radaković SS Šurbatović M Radaković A Pavlica M. Nutrigenetics—the role of nutrition and heritage in the development and prevention of malignant disease. Vojnosanit Pregl. (2004) 61:65–70. 10.2298/VSP0401065R 15022391

[15] Cooke MS Evans MD Dizdaroglu M Lunec J. Oxidative DNA damage: mechanisms, mutation, and disease. FASEB J. (2003) 17:1195–214. 10.1096/fj.02-0752rev 12832285

[16] Jaffe M Uber den Niederschlag welchen Pikrinsaure in normalem Harn erzeugt und uber eine neue Reaction des Kreatinins. Z Physiol Chem. (1886) 10:391–400.

[17] Han W Yu KN Ionizing radiation, DNA double strand break and mutation. In: Urbano KV, editor. Advances in Genetics Research. New York, NY: Nova Science Publishers, Inc (2010). p. 1–13.

[18] Kryston TB Georgiev AB Pissis P Georgakilas AG. Role of oxidative stress and DNA damage in human carcinogenesis. Mutat Res. (2011) 711:193–201. 10.1016/j.mrfmmm.2010.12.016 21216256

[19] Tucker JD Vdapalli M Joiner MC Ceppi M Fenech M Bonassi S. Estimating the lowest detectable dose of ionizing radiation by the cytokinesis-block micronucleus assay. Radiat Res. (2013) 180:284–91. 10.1667/RR3346.1 23931722

[20] Mrdjanovic J Jakimov D Tursijan S Bogdanovic G. Evaluation of sister chromatide exchanges, micronuclei, and proliferate index in hospital workers chronically exposed to ionizing radiation. J BUON. (2005) 10:99–105. 17335139

[21] Fenech M Kirsch-Volders M Natarajan AT. Molecular mechanisms of micronucleus, nucleoplasmic bridge and nuclear bud formation in mammalian and human cells. Mutagen. (2010) 26:125–32. 10.1093/mutage/geq052 21164193

[22] Pala FS Alkaya F Tabakçioglu K Tokatli F Uzal C Parlar S The effects of micronuclei with whole chromosomes on biological dose estimation. Turk J Biol. (2008) 32:283–90.

[23] Petkau A. Role of superoxide dismutase in modification of radiation injury. Br J Cancer. (1987) 8:87. 3307878 PMC2149491

[24] Neill PO Wardman P. Radiation chemistry comes before radiation biology. Int J Radiat Biol. (2009) 85:9–25. 10.1080/09553000802640401 19205982

[25] Evans MD Olinski R Loft S Cooke MS. European Standards Committee on Urinary (DNA) Lesion Analysis Toward consensus in the analysis of urinary 8-oxo-7, 8-dihydro-2′-deoxyguanosine as a noninvasive biomarker of oxidative stress. FASEB J. (2010) 24:1249–60. 10.1096/fj.09-147124 19966135 PMC6188230

[26] Mrdjanovic J Jungic S Solajic S Bogdanovic V Jurišic V Effects of orally administered antioxidants on micronuclei and sister chromatid exchange frequency in workers professionally exposed to antineoplastic agents. Food Chem Toxicol. (2012) 50:2937–44. 10.1016/j.fct.2012.04.027 22546365

[27] Mrdjanovic J Šolajic S Dimitrijevic S Dan I Nikolic I Jurišic V. Assessment of micronuclei and sister chromatid exchange frequency in the petroleum industry workers in province of Vojvodina, Republic of Serbia. Food Chem Toxicol. (2014) 69:63–8. 10.1016/j.fct.2014.03.041 24721434

[28] Fenech M Bonassi S. The effect of age, gender, diet and lifestyle on DNA damage measured using micronucleus frequency in human peripheral blood lymphocytes. Mutagen. (2011) 26:43–9. 10.1093/mutage/geq050 21164181

[29] Fenech M Neville S Rinaldi J. Sex is an important variable affecting spontaneous micronucleus frequency in cytokinesis-blocked lymphocytes. Mutat Res Environ Mutag Relat Subjects. (1994) 313:203–7. 10.1016/0165-1161(94)90050-7 7523905

[30] Zanolin ME Girardi P Degan P Rava M Olivieri M Di Gennaro G. Measurement of a urinary marker (8-hydroxydeoxy-guanosine, 8-OHdG) of DNA oxidative stress in epidemiological surveys: a pilot study. Int J Biol Markers. (2015) 30:e341–5. 10.5301/jbm.5000129 25588860

[31] Kulikowska-Karpinska E Czerw K. Estimation of 8-hydroxy-2-deoxyguanosine (8-OHdG)concentration in the urine of cigarette smokers. Wiad Lek. (2015) 68:32–8. 26094331

[32] Rana SVS Verma Y Singh GD. Assessment of genotoxicity amongst smokers, alcoholics, and tobacco chewers of North India using micronucleus assay and urinary 8-hydroxyl-2′-deoxyguanosine, as biomarkers. Environ Monit Assess. (2017) 189:391. 10.1007/s10661-017-6103-3 28702879

